# Children with congenital colorectal malformations during the UK Sars-CoV-2 pandemic lockdown: an assessment of telemedicine and impact on health

**DOI:** 10.1007/s00383-021-04971-6

**Published:** 2021-08-05

**Authors:** E. Stathopoulos, C. Skerritt, G. Fitzpatrick, E. Hooker, A. Lander, O. Gee, I. Jester

**Affiliations:** Department of Paediatric Surgery, Birmingham Women’s and Children’s Hospital, Steelhouse Lane, Birmingham, UK

**Keywords:** Anorectal malformation, Hirschsprung’s disease, Quality of life, Sars-CoV-2, COVID-19, Telemedicine

## Abstract

**Purpose:**

This pilot study was designed to assess bowel function and quality of life (QoL) in children and adolescents with congenital colorectal malformations (CCM) during the first UK COVID lockdown period.

**Methods:**

Changes in health were assessed through semi-structured interviews, gastrointestinal functional outcomes using Krickenbeck scoring and QoL by the modified disease-specific HAQL (Hirschsprung’s disease anorectal malformation quality of life questionnaire). The State-Trait Anxiety Inventory (STAI)™ for adults was used to assess parental anxiety.

**Results:**

Thirty-two families were interviewed; 19 (59%) reported no change in their child’s health during the lockdown, 5 (16%) a deterioration and 8 (25%) an improvement. Neither the severity of the CCM, nor the degree of bowel dysfunction, correlated with any deterioration. The HAQL score was not correlated to a change in health. Anxiety scores ranged from no anxiety to clinical concerns. Telemedicine was well accepted by 28/32 parents (88%); however, in-person appointments were preferred if there were clinical concerns.

**Conclusion:**

In the follow-up of children and adolescents with CCM during the first UK lockdown using telemedicine we found that over half had stable health conditions. Patients needing additional care could not be predicted by the severity of their disease or their bowel function alone.

**Supplementary Information:**

The online version contains supplementary material available at 10.1007/s00383-021-04971-6.

## Introduction

The arrival of Sars-CoV-2, the coronavirus responsible for the current pandemic, forced hospitals and health care systems to reorganize services for many patients. Non-urgent appointments were rescheduled, and telemedicine was rapidly implemented.

The national lockdown imposed by the UK government on March 23rd, 2020 meant that schools were closed for all children, apart from those of key workers. Children’s lives were disrupted in an unprecedented way.

Children born with a congenital colorectal malformation (CCM), such as Hirschsprung’s disease (HSCR) or anorectal malformations (ARM), are followed at our institution by a dedicated team of surgeons and nurses. Our children’s colorectal clinic became virtual in line with our hospital’s pandemic response, offering telephone or video consultations.

Constipation and fecal incontinence are known long-term complications in both mild and severe forms of congenital colorectal malformations [1, 2]. The congenital colorectal clinic aims to monitor and alleviate abnormal bowel function, using a bowel management program [3].

We hypothesized that lockdown and modified access to health care might alter the bowel function and the quality of life of children and adolescents with CCM. Therefore, the study’s main aim was to assess bowel function and quality of life of children and adolescents with CCM during the lockdown. Two secondary objectives were to (1) assess the parental anxiety and (2) collect the opinions of parents regarding telemedicine.

## Patients and methods

### Participants

Forty patients aged 5–16 years with CCM operated on and followed at the congenital colorectal clinic at Birmingham Women’s and Children’s Hospital, who had an appointment scheduled during the lockdown period (April–May 2020), were asked to participate by their surgeon (IJ, OG, AL) or the colorectal nurses (GF, EH). During the first UK lockdown period, regular outpatients’ appointments were replaced by telephone clinics at our institution and no patient was seen face to face except in emergency situation. Lack of English proficiency was an exclusion criterion.

### Severity of the malformation

The severity of an ARM was classed as mild (perineal and vestibular fistula in girls, perineal fistula in boys) and severe (cloaca and recto-vaginal fistula in girls, recto-urethral fistula in boys). In HSCR, severity depended on the aganglionic segment length: mild was rectosigmoid aganglionosis, and severe was long-segment or total colonic aganglionosis.

### Semi-structured interviews

Semi-structured interviews were conducted over the phone by two authors (ES, CS), who were not involved in patient care. Two dimensions were explored: parental perception of their child’s health related to the colorectal disease and access to health care. The questions are summarized in Online Appendix 1.

### Assessment of digestive functional outcome

Functional outcome was assessed following the Krickenbeck scoring system (see Online Appendix 2). Participants managed with retrograde colonic enemas or antegrade enemas were excluded from this assessment.

### Quality of life assessment

Quality of life was assessed using a disease-specific questionnaire, the modified HAQL (Hirschsprung’s disease anorectal malformation quality of life questionnaire) [4, 5]. There are three age-specific versions of the questionnaires (6–11, 12–16, > 17 years); proxy questionnaires exist for two younger age groups. Seven dimensions that cover physical, emotional and social functioning, as well as disease-related symptoms, are explored by multiple items: the presence of diarrhea (4 items), fecal continence (6 items), physical symptoms (8 items), emotional functioning (9 items), use of laxative diet (2 items), urinary continence (4 items), and social functioning (3 items). For patients with a stoma, extra items (8) related to the stoma are included and items related to bowel motions omitted. Responses are scored (0–3) depending on the frequency (never, sometimes, often, very often) of the specific problem during the past 7 days. The values are transformed into a score for each dimension (0–100) which are summed for all seven dimensions (0–700).

There are validated and culturally adapted French, Dutch, Swedish and Italian versions; an English translation exists but has not been validated.

Questions were administered over the phone by two authors (ES, CS) not involved in patient care. The questionnaire has not been validated for those with developmental delay or a cloaca. Such patients were included in our study, and their results are reported separately.

### Assessment of parental anxiety

Anxiety in a parent or primary carer was assessed using the State-Trait Anxiety Inventory (STAI)™ for adults. The STAI consists of two questionnaires of 20 items each: the first questionnaire (state anxiety) measures how the subject feels at a particular moment in time, the second (trait anxiety) how they feel generally. Examples of items are “I feel at ease”, “I feel upset”, “I am a steady person”. The psychometric properties of both questionnaires are well-established [6]. After gaining consent, the questionnaires were sent electronically or by mail.

### Statistical analysis

Demographical data are presented descriptively. Average HAQL scores are reported as mean ± standard deviation. Student’s *t* test was used to look for difference in HAQL scores according to severity of malformation, presence of soiling or constipation or a deterioration in health. Statistical analyses were performed using GraphPad Prism 9.0.0 (GraphPad Software, San Diego, CA, United States).

## Results

### Population characteristics

Out of the 40 patients identified, 32 [21 boys (67%), 11 girls (33%)] participated, 5 (13%) accepted to participate but did not answer our subsequent calls and 3 withdrew (8%). Eleven children had Hirschsprung’s disease, of whom 4 (36%) had long segment/total colonic aganglionosis. Twenty-one children had an anorectal malformation, of whom 10 (48%) had severe malformations including 2 patients with a cloaca. Therefore, according to the predetermined classification 18/32 (56%) of children had congenital colorectal conditions of mild severity and 14/32 (44%) were classified as severe.

Fourteen (44%) patients had other co-morbidities (see Table 1). Ten (31.5%) children were managed with mechanical irrigation via ACE stomas or transrectally. A further four (12.5%) children had stomas (3 colostomies, 1 ileostomy). Four (12.5%) children had developmental delay.

**Table 1:** Demographics

Demographics	Mild HSCR (*n* = 7)	Severe HSCR (*n* = 4)	Mild ARM (*n* = 11)	Severe ARM (*n* = 10)	Total (*n* = 32)
Male:female	6:1	2:2	5:6	8:2	21:11
Isolated condition	5 (71%)	1 (25%)	7 (64%)	5 (50%)	18 (56%)
Co-morbidities	2 ADHD	1 Short gut 1 Chromosomal (T21) 1 Deaf	2 Chromosomal (T21, Di George) 1 Urological 1 ADHD	1 VACTERL 1 VACTERL + chromosomal 2 Urological 1 Haem	14 (44%)
Developmental delay	0	1 (25%)	2 (18%)	1 (10%)	4 (12.5%)
Enema/ACE	1 (14%)	0	4 (36%)	5 (50%)	10 (31%)
Stoma	2 (29%)	2 (50%)	0	0	4 (12.5%)

### Telemedicine

During the first UK lockdown period, all 32 patients were followed by telephone clinics as scheduled. No patient presented to an Emergency department.

Fourteen out of 32 (44%) parents preferred virtual clinics, 4 (12%) parents stated they disliked them, and 14 (44%) felt they were as good as face-to-face clinics. All parents who disliked telephone clinics have a child with a severe ARM. Among the 28 parents who were satisfied with virtual clinics, 16 (57%) spontaneously commented that they would like their child to be seen face to face if there was a problem.

### Health during lockdown

In our group of patients, 19 out of 32 (59%) reported no change in their child’s health during lockdown while 8 out of 32 (25%) reported an improvement and 5 out of 32 (16%) commented that their child’s health had deteriorated. There was no correlation between the severity of the congenital colorectal malformation, the degree of bowel dysfunction and the deterioration in the child’s health (Table 2). There was no correlation with the overall HAQL score and the report of a deterioration in the child’s health (Table 3).

**Table 2:** Survey results according to severity and type of colorectal condition

	Mild HSCR (*n* = 7)	Severe HSCR (*n* = 4)	Mild ARM (*n* = 11)	Severe ARM (*n* = 10)	Total (*n* = 32)
Change in Health					
Improved	1	1	4	2	8 (25%)
Deteriorated	2	0	1	2	5 (16%)
No change	4	3	6	6	19 (59%)
Telemedicine					
Preferred	4	2	5	3	14 (44%)
Disliked	0	0	0	4	4 (12%)
Neutral	3	2	6	3	14 (44%)
Krickenbeck Scoring	*n* = 4	*n* = 2	*n* = 6	*n* = 5	*n* = 17
Soiling					
Grade 0	3	1	3	2	9 (53%)
Grade 1	0	0	2	2	4 (24.5%)
Grade 2	1	1	1	1	4 (24.5%)
Grade 3	0	0	0	0	0
Constipation					
Grade 0	2	1	3	2	8 (47%)
Grade 1	0	0	2	0	1 (6%)
Grade 2	2	1	1	2	7 (41%)
Grade 3	0	0	0	1	1 (6%)
HAQL (mean, sd)	*n* = 7 539 (± 112)	*n* = 4 611 (± 41) Without DD *n* = 3 609 (± 50)	*n* = 8 617 (± 106) Without DD *n* = 6 616 (± 124)	*n* = 6 617 (± 58) Without cloaca *n* = 4 620 (± 58)	*n* = 25 594 (± 92) Without DD *n* = 22 591 (± 98) Without DD OR cloaca *n* = 20 588 (± 101)

**Table 3:** HAQL scores according to the severity of the malformation, presence of ACE/stoma, presence of soiling/constipation and deterioration in health

	Yes	No	*p* value
Severe malformation	614 (50)	581 (112)	0.388
Presence of ACE/stoma	551 (114)	628 (55)	0.035*
Soiling	599 (50.3)	657 (48)	0.057
Constipation	601 (40)	647 (62)	0.147
Deterioration in health	593 (61)	591 (99)	0.97

Mean (standard deviation)

*statistically significant

Parents repeatedly stated that the reason for an improvement in their child was (i) that they had better control of their child’s diet, (ii) better control of their child’s schedule (including the timing of stooling) and (iii) the absence of peers’ judgment. Less physical exercise and less social contact were the reasons given to explain health deterioration during the lockdown.

### Functional assessment

Seventeen out of eighteen (94%) children who did not have either an ACE or a stoma were assessed with the Krickenbeck assessment tool. In our sample of patients, 9 (53%) had no soiling, and 8 (47%) had no constipation (Table 2).

### Quality of life

Out of 32 patients, 25 (78%) patients’ quality of life was assessed with the HAQL; four patients did not complete the questionnaire, one child had such severe developmental delay that questions in the social dimension were not applicable and the parents of two children who were 6 and 7 years old respectively did not feel that they were able to answer questions in the social dimension due to their young age. All but one of the questionnaires was answered by a parent.

The mean (± SD) score was 594 (± 92) from a maximum of 700. In our group, quality of life appeared independent of the malformation severity (Table 3). An ACE or stoma were the only factors that significantly negatively affected a child’s quality of life, *p* = 0.035. Constipation did not appear to affect the quality of life. There was a trend towards a worse quality of life in children who soiled, although this did not reach statistical significance, *p* = 0.057.

The HAQL score was not related to a reported deterioration in a child’s health.

### Parental anxiety

Eight parents (24%) returned the STAI questionnaire. The range of anxiety scores was from no anxiety to clinical concerns. Parents of children with a severe congenital colorectal malformation had higher trait anxiety. There was no difference based on the type of malformation, the presence of colostomy or ACE (Table 4).

**Table 4:** STAI state and trait assessment in our group (*n* = 8)

	Mild (*n* = 3)	Severe (*n* = 5)	ARM (*n* = 4)	HSCR (*n* = 4)	With colostomy (*n* = 2)	No colostomy (*n* = 6)	With ACE (*n* = 2)	No ACE (*n* = 6)
STAI-State	40 (14)	31 (17)	39 (15)	30 (17)	22 (3)	38 (16)	29 (0)	36 (18)
STAI-Trait	41 (4)	27 (4)	34 (6)	30 (10)	27 (1)	34 (8)	37 (5)	31 (8)

Mean (standard deviation)

## Discussion

Our study of health changes in children with CCMs during the UK Sars-CoV-2 lockdown showed no change in 19 out of 32 (59%) according to parental reporting; 5 out of 32 (16%) reported a deterioration and 8 out of 32 (25%) an improvement. There was no correlation between the severity of the colorectal malformation or the quality of life assessment and the report of a change in the child’s health condition. Parents linked improvement with a better diet, a more regular stooling timing, and an absence of peers’ judgment; deterioration was attributed to decreased physical exercise and social contacts. Anxiety among carers ranged from no anxiety to pathological levels suggesting that they would benefit from targeted medical management.

The absence of a clear link between bowel function and health perception in our study mirrors the fact that the effect of fecal incontinence or constipation on the quality of life of children with congenital colorectal malformation remains unclear. In the mild form of ARM, Grano et al. found that fecal incontinence negatively affected various dimensions of adolescent quality of life [7]. However, studying a comparable population of children with mild ARM, Wigander et al. did not show any correlation between functional problems and quality of life [8]. Similarly, follow-up studies of quality of life in HSCR patients showed equivocal results. Variable degrees of functional impairment were found in pediatric and adult patients and were not always associated with altered overall quality of life when compared to healthy controls [9, 10]. However, studies of HSCR patients with total colonic aganglionosis have shown a negative impact on quality of life compared with healthy peers [11].

The absence of a relationship between disease severity, bowel function, and quality of life makes it difficult for clinicians to predict the patients and parental needs. In a standard setting, Hartman et al. suggested that patients’ perception of the disease and their life coping strategies were as important as disease severity and additional congenital anomalies, in terms of their influence on quality of life [12]. Our findings illustrate that identifying patients at risk for health problems cannot be identified by relying solely on disease severity, bowel function or quality of life and inquiry into psychosocial resources should be sought.

Social relations constitute one of the domains of the quality of life as defined by the World Health Organization. Bowel dysfunction may influence the health-related quality of life of patients with CCMs. In a series of 33 adolescents with ARMs, Diseth and Emblem found that incontinence to flatus or stool was associated with psychiatric or psychosocial impairment [13]. However, Ojmyr-Joelsson et al. described a series of 25 school-aged children with severe ARMs who reported either constipation or fecal incontinence, and did not show lower emotional health or self-esteem and reported good relationships with their friends [14]. During the time of our study, social relationships were turned upside down due to the lockdown. Some parents perceived this as a benefit to their child’s health, for example, if their child experienced bullying at school; however, for other children a lack of social interaction was a hindrance to their well-being.

Our study has several notable limitations. The sample size is small and not representative of the actual distribution of CCMs. Patients with a severe form of ARM or HSCR constitute a larger proportion of our cohort when compared to the expected distribution [15, 16]. Likewise, patients using an ACE for either fecal incontinence or constipation are overrepresented in our cohort. This selection bias may stem from the fact that participants were selected during a short period through medical outpatient appointments and nurse-led clinics—where there is a focus on maintaining adequate washout and stoma bag supplies for patients. However, this selection bias allowed us to have a qualitative assessment of a broad spectrum of children with congenital colorectal malformations and associated anomalies, ranging from patients doing well with minimal intervention to those in a structured bowel management program.

Secondly, the outcomes are, for the most part, reported by the parents and may not reflect the child’s or the adolescent’s point of view. Although the adolescent or the older child was allowed to express themselves, the vast majority declined.

The third important limitation is the lack of functional and quality of life scores gathered during the period preceding the lockdown. Therefore, no comparison can be drawn in absence of a reference score.

Telemedicine in pediatric surgery was not considered the usual standard of care [17]. During the Sars-CoV-2 pandemic, most of our patients with chronic health conditions have been managed by telemedicine. However, this delivery of pediatric surgical services by telemedicine has not been formally assessed. In a survey of parental perception of telemedicine in a pediatric population, Abdulhai et al. showed that it was supported by 70% of the respondents regarding postoperative visits and 68% for subspecialty evaluations [18]. In our study it was only possible to perform telephone clinics. Despite this, 88% of the parents were happy and satisfied to have a telemedicine follow-up. Fifty-seven percent wanted to see their physician if they experienced a problem. It can be assumed that this high percentage might improve by use of video clinics. Unfortunately, this technology did not yet exist at the beginning of the first lockdown in our hospital in 2020.

Despite its limitations, this study allowed us to have a broad look at our patients’ health during the lockdown and investigate telemedicine’s efficacy. A more extensive cohort study would be required to confirm these preliminary results and inform a change in practice to focus on the patient and parent-reported quality of life as part of routine care. The evidence of psychosocial concerns influencing quality of life suggests that many of these patients would benefit from more structured psychological support.

## Conclusion

Severity of the CCM or degree of bowel dysfunction are not linked to deterioration of health during the Sars-CoV-2 pandemic induced lockdown period. Follow-up of these children and adolescents by telemedicine is possible but the assessment should contain both disease-specific and psychosocial questions to allow detection of patients at risk for quality of life problems.

## Supplementary Information

Below is the link to the electronic supplementary material.

Supplementary file1 (DOCX 12 kb)
