# Supplementary material for: Children with congenital colorectal malformations during the UK Sars-CoV-2 pandemic lockdown: an assessment of telemedicine and impact on health
Source: Pediatr Surg Int. 2021 Aug 5;37(11):1593–9. doi: 10.1007/s00383-021-04971-6 (PMC8340806; doi:10.1007/s00383-021-04971-6)
Supplement: Supplementary file 1 — Supplementary file1 (DOCX 12 kb) [file 383_2021_4971_MOESM1_ESM.docx]

SUPPLEMENTAL MATERIAL

Appendix 1. Semi-structured interview.

Exploration of child’s health condition:

*Do you think the health condition of your child has changed during lockdown?*

*If yes, has it improved or deteriorated?*

*Could you explain to us why, in your opinion, their health condition has changed?*

Opinion about telephone clinic:

*Could you ask your questions during the telephone clinic?*

*Does it help answer your current questions or problem?*

*What do you think about the safety of telephone clinic?*

*What do you think about telephone clinic as a follow up mean?*

Appendix 2. Krickenbeck assessment tool.

| **Voluntary bowel movements** | Yes/No |
| --- | --- |
| Feeling the urge | Yes/No |
| **Soiling** | Yes/No |
| Grade 1 | Occasionally (one/twice per week) |
| Grade 2 | Every day, no social problem |
| Grade 3 | Constant, social problem |
| **Constipation** | Yes/No |
| Grade 1 | Manageable by change in diet |
| Grade 2 | Require laxative |
| Grade 3 | Resistant to laxative and diet |
